# Influenza seasonality in Madagascar: the mysterious African free-runner

**DOI:** 10.1111/irv.12308

**Published:** 2015-04-23

**Authors:** Wladimir Jimenez Alonso, Julia Guillebaud, Cecile Viboud, Norosoa Harline Razanajatovo, Arnaud Orelle, Steven Zhixiang Zhou, Laurence Randrianasolo, Jean-Michel Heraud

**Affiliations:** a Fogarty International Center, National Institutes of HealthBethesda, MD, USA; b National Influenza Center, Virology Unit, Institut Pasteur of MadagascarAntananarivo, Madagascar; c Integrated Quality Laboratory Services (IQLS)Lyon, France; d London School of Hygiene & Tropical MedicineLondon, UK; e Epidemiology Unit, Institut Pasteur of MadagascarAntananarivo, Madagascar

**Keywords:** Influenza, Madagascar, population connectivity, seasonality, time series, viral migration

## Abstract

**Background:**

The seasonal drivers of influenza activity remain debated in tropical settings where epidemics are not clearly phased. Antananarivo is a particularly interesting case study because it is in Madagascar, an island situated in the tropics and with quantifiable connectivity levels to other countries.

**Objectives:**

We aimed at disentangling the role of environmental forcing and population fluxes on influenza seasonality in Madagascar.

**Methods:**

We compiled weekly counts of laboratory-confirmed influenza-positive specimens for the period 2002 to 2012 collected in Antananarivo, with data available from sub-Saharan countries and countries contributing most foreign travelers to Madagascar. Daily climate indicators were compiled for the study period.

**Results:**

Overall, influenza activity detected in Antananarivo predated that identified in temperate Northern Hemisphere locations. This activity presented poor temporal matching with viral activity in other countries from the African continent or countries highly connected to Madagascar excepted for A(H1N1)pdm09. Influenza detection in Antananarivo was not associated with travel activity and, although it was positively correlated with all climatic variables studied, such association was weak.

**Conclusions:**

The timing of influenza activity in Antananarivo is irregular, is not driven by climate, and does not align with that of countries in geographic proximity or highly connected to Madagascar. This work opens fresh questions regarding the drivers of influenza seasonality globally particularly in mid-latitude and less-connected regions to tailor vaccine strategies locally.

## Introduction

The biological mechanisms responsible for the seasonality of influenza and other respiratory infections remain to this day one of the most intriguing topics in infectious disease epidemiology. Influenza predominates in winter in temperate regions of the world and in the rainy season in the Tropics,^1^–^3^ although many tropical and subtropical regions experience more complex seasonal patterns including semiannual cycles and year-round activity.^3^–^5^ Many putative mechanisms have been put forward, but a unifying theory accounting for the diversity of seasonal patterns observed globally remains elusive.^3^,^6^–^8^ A better understanding of influenza seasonality is important to help tailor surveillance strategies and optimize the local timing of influenza vaccination,^9^ especially in developing country settings where influenza disease burden is becoming increasingly recognized.^10^–^14^

The complicated web of existing theories addressing influenza seasonality can be categorized in three types of mechanisms related to virus survival, host immunity, and contact rates.^3^ Each of these mechanisms might be subject to the influence of a series of seasonal stimuli including humidity, solar radiation, temperature, nutrition, precipitation, and socio-behavioral factors.^3^ One of the difficulties in gathering evidence for a specific mechanism is that a single seasonal stimulus can theoretically act in more than one mechanism. For instance, it has been proposed that low temperature enhances virus survival, lowers host immunity, and increases host contact rate (as people may spend more time indoors in winter).^7^,^15^ Moreover, in settings where seasonal fluctuations in climate factors are limited, other factors can then dictate the pace of influenza infections, including population fluxes with regions that experience more seasonal outbreaks.^7^ Indeed, the seasonality of influenza epidemics has shifted with increased global connectivity, as shown in historical records from Iceland and recent surveillance data from southern China.^16^,^17^ Further, the onset of the first wave of pandemic influenza A(H1N1)pdm09 in the United Kingdom (UK) can be linked to travel fluxes from the United States of America (USA) and Mexico compounded by the timing of school vacation.^18^ Although randomized controlled trials and laboratory experiments will probably provide the strongest evidence to support one theory or the other, epidemiological studies in a variety a globally sampled location experiencing diverse environmental and demographic conditions can help put forward specific hypotheses.^7^

Madagascar provides a unique case study because it is situated in the tropical belt and is an island, and therefore, external virus seeding is presumably more limited than elsewhere. Most importantly, there is a long tradition of monitoring influenza virus activity at the National Influenza Center (NIC) located at the Institut Pasteur of Madagascar, allowing for robust multiyear epidemiological studies.^14^,^19^–^22^ In this modeling study, we attempt to disentangle the role of environmental forcing and population fluxes on influenza seasonality in Madagascar. Specifically, we compare the seasonal patterns of influenza virus activity in Antananarivo (the capital city) to that of other areas that have important geographic or travel connections to Madagascar.

## Methods

### Study site

Antananarivo is the capital city of Madagascar, which is located in the central highlands at 1280 m of altitude. The subtropical city, located at 18·55′S latitude and 47·32′W longitude, is characterized by mild, dry winters, and warm and rainy summers. The city includes administrative, commercial, industrial, and residential areas, interspaced with patches of agricultural land (mainly rice fields). The city population size was about 2 million in 2012.

### Influenza data in Antananarivo

Influenza surveillance in Madagascar is a long-standing laboratory-based effort based at the Institut Pasteur of Madagascar (NIC).^23^,^24^ In Antananarivo, this surveillance relies on a network of six sentinel primary healthcare centers. Each participating sites reports the number of influenza-like illnesses (ILI) meeting the modified WHO standard case definition,^14^ and the total number of patient visits for each reporting day (Monday to Friday). Respiratory specimens are collected by health worker staffs and shipped to the NIC to be tested for the presence influenza virus by viral isolation (before 2009) and RT–PCR (since 2009), as previously described.^25^ We compiled weekly counts of influenza A(H3N2), seasonal A(H1N1), A(H1N1)pdm09, and B-positive specimens and the number of specimen tested for the period 2002–2012. Because of the sampling differences through years, we rescaled the data per year (from zero to one).^26^

### Travel data

To obtain information on locations that are well connected with Madagascar and are potentially important drivers of influenza virus circulation in the island, we compiled annual data on the number of visitors to Madagascar from the Ministry of Tourism for the period 2002–2012. The top seven countries contributing the greatest volume of visitors to Madagascar in the period from 2002 to 2012 included France (56% of annual travelers ±2%), La Réunion island (16 ± 3%), Italy (6 ± 1%), US (4 ± 1%), Germany (4 ± 1%), United Kingdom (3 ± 1%), Switzerland (2 ± 1%), and others (16 ± 3%) (Figure S1). The greatest influx of foreign visitors typically occurred in the second semester of the year, with greatest volume observed in July (winter period in Madagascar, summer period in Europe). We used this information as a proxy of international population mixing, to infer which countries were the most likely origins of influenza virus introductions into Madagascar during our study period.

### Temporal comparison with influenza activity in other countries

To compare influenza activity in Antananarivo with that of other countries, we downloaded weekly time series of laboratory-confirmed influenza cases by virus type (seasonal A(H1N1), A(H1N1)pdm09, A(H3N2), A(unsubtyped), and B), from FluNet, the global influenza surveillance system maintained by WHO (http://www.who.int/influenza/gisrs_laboratory/flunet/en). We used influenza information for the following set of countries:

1. Countries that contributed the most travelers to Madagascar include France, Mauritius (an island geographically close to La Réunion, which is well connected to Madagascar but where influenza data were not available), Italy, USA, Germany, UK, and Switzerland.
2. Countries in sub-Saharan Africa that are relatively close geographically to Madagascar and contribute sufficient influenza surveillance data to allow comparison: Democratic Republic of the Congo (DRC), Kenya, Nigeria, Senegal, South Africa, and Uganda. These countries were selected based on data available on FluNet and in a special issue of the Journal of Infectious Diseases dedicated to “Influenza in Africa” (http://jid.oxfordjournals.org/content/206/suppl_1.toc).

We arbitrarily decided to exclude from analysis, countries that did not have a minimum of 50 samples in the period from March 2009 to July 2010 (a period of high sampling intensity indicative of the strength of national surveillance systems).

### Climatology

Daily climatologic data were extracted for Antananarivo from the database from the weather underground website (http://english.wunderground.com/history/airport/FMMI/CustomHistory.html) and from the International Research Institute for Climate and Society (http://iridl.ldeo.columbia.edu/). Data were averaged by week for temperature, dew point, and humidity (for temperature and humidity, the maximum and minimum values per week were also used as additional variables). Data were available for the whole period of interest (2002–2012), except for precipitation, which was only available from 2006 to 2011. We excluded January 2004 from the analysis as climate data was missing for this month.

### Statistical analyses

Firstly, we sought to identify periodic cycles (i.e., seasonal patterns) in the Antananarivo influenza time series using Windowed Fourier analysis as previously described.^27^ We summed up the 12-monthly, 6-monthly, and 3-monthly harmonics obtained by Fourier analysis to create a “periodic annual function” (PAF). This PAF has the seasonal signature of the original data, where year-to-year variations (trends and anomalies) are removed, but seasonal variations within the year are preserved. Further, we compared the seasonality between Antananarivo and epidemiologically relevant locations in a nonparametric fashion. We applied a hierarchical clustering approach to whole time series (using average linkage method, relying on the inverse of the correlation coefficients between the time series as the distance metrics), which helped identify influenza epidemiological similar “regions”.^26^,^28^ Those analyses and the visual representation of the series in heat grids were performed using the Epipoi freeware.^29^

To explore the potential role of climate, we calculated pairwise Spearman correlation coefficients between standardized influenza time series and climate indicators. Correlations were calculated for the entire study period after exclusion of year 2009 (as this was an atypical pandemic year) and January 2004 (due to missing climate data) and also stratified by semester, considering the 1st half of the year (weeks 1 through 26) versus the 2nd half (weeks 27–52), and the colder season (weeks 13–38) versus the warmer season (weeks 39–12). Correlations were computed for lags ranging between 0 and 5 weeks in climate data.

## Results

### Influenza virus activity in Antananarivo, Madagascar, from 2002 to 2012

**Figure 1:**
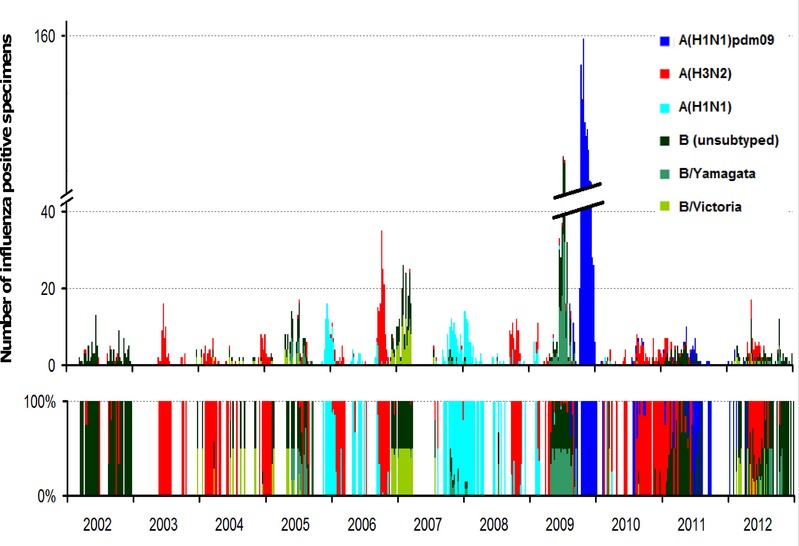
Weekly detection of influenza viruses in Antananarivo, Madagascar 2002–2012. Upper panel: counts of positive specimens by subtype; lower panel: relative contribution of each subtype.

Weekly patterns of influenza virus activity in Antananarivo between 2002 and 2012 are illustrated in Figure1. Reports of virus activity intensified during 2009, which is mostly due to the strengthening of the surveillance system due to the pandemic. Influenza B predominated in the middle of the 2009, and A(H1N1)pdm09 did not become dominant until October 2009.^25^ In interpandemic years, different subtypes of influenza cocirculated without any noticeable periodicity pattern.

**Figure 2:**
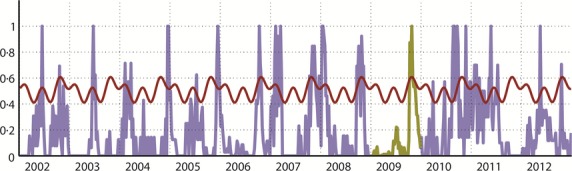
Time series of weekly detection of influenza standardized from zero to one per year in Antananarivo and seasonal model based on Fourier decomposition 29 from 2002 to 2012. The red line shows the upper bound of the 95 percent confidence interval (95% CI) of the periodic annual function obtained by the sum of the three first harmonics, indicating three peaks per year. Data for year 2009 were excluded from model building (highlighted in green) because this was anomalous year (influenza activity was much higher than usual due to the circulation of the pandemic virus A(H1N1)pdm09).

Visual inspection of the time series does not reveal a clear seasonal pattern. Some years reveal the presence of two influenza peaks in different semesters (2002, 2004 to 2008, 2012, and even 2009, although this was a non-typical influenza season). In contrast, in other years, we can distinguish only one clear influenza peak (2003, 2010, and 2011). Accordingly, the average seasonal signature (PAF) of the yearly standardized (from zero to one) influenza in Antananarivo, excluding the pandemic year of 2009, shows an almost flat periodic annual signal (Figure2). Hence, Fourier decomposition supports a lack of influenza seasonality in Antananarivo.

### Temporal comparison with influenza circulation in other countries

**Figure 3:**
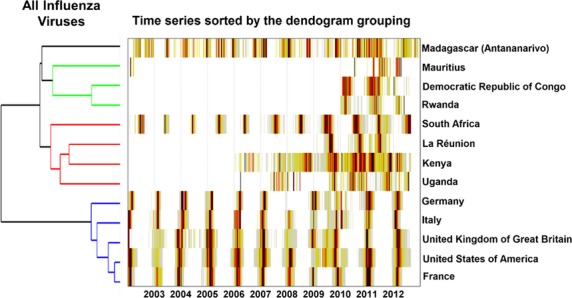
Heat-grid and hierarchical clustering of total influenza viruses detected in Antananarivo and in countries in geographic proximity or linked by travel to Madagascar.

We used hierarchical clustering to identify the countries most similar to Antananarivo with respect to influenza circulation. Comparison of influenza patterns in Antananarivo and geographically close or well-connected locales revealed three epidemiologically relevant groups, as shown in Figure3. The first group comprised European countries and the USA, where influenza virus circulation is concentrated in the Northern Hemisphere winter months, December through March. A second group represented select African countries, including South Africa, La Réunion, Kenya, and Uganda, where influenza peaks typically coincide with the Southern Hemisphere winter (a pattern most pronounced for South Africa, and least pronounced for Uganda, possibly due to sampling issues^30^). Antananarivo clustered with a 3rd group of African countries, including Mauritius, DRC, and Rwanda, which were characterized by year-round influenza activity or peaks in March–May, intermediate between the Northern and Southern Hemisphere winters.

**Figure 4:**
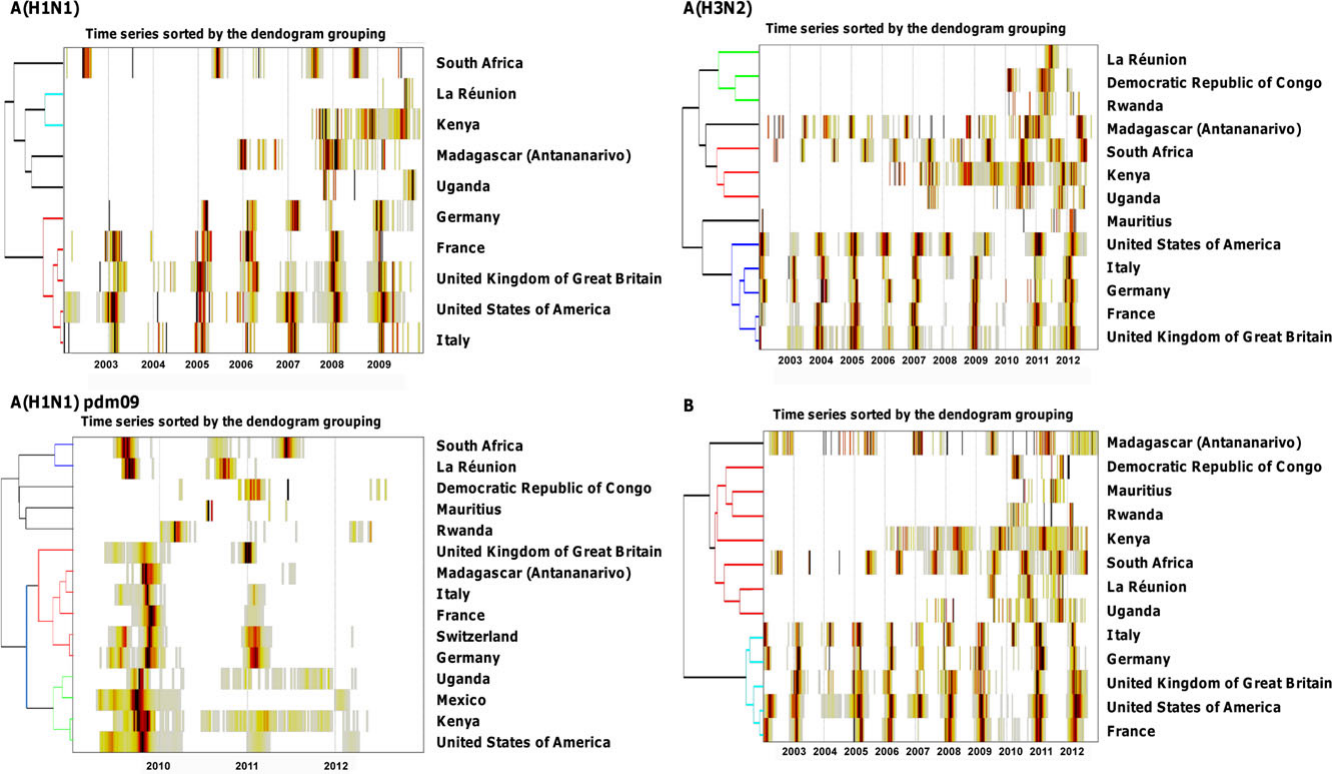
Heat-grid and hierarchical clustering of seasonal influenza A(H1N1), A(H3N2), A(H1N1)pdm09, and B viruses detected in Madagascar (Antananarivo) and in countries in geographic proximity or linked by travel to Madagascar.

Next, we repeated the hierarchical clustering analysis for each virus subtype separately (Figure4). The influenza clusters identified differed depending on virus type, although the group representing Europe and the USA was stable in analyses of influenza A(H3N2), seasonal A(H1N1), and B. In contrast, the position of Antananarivo in the hierarchical tree varied substantially between influenza subtypes, as detailed below.

Seasonal A(H1N1) was episodically detected since 2006 in Antananarivo, with a single widespread epidemic in the first semester of 2008 (Figure4). Circulation of seasonal A(H1N1) in Antananarivo preceded that in the Northern Hemisphere countries by a few weeks and was completely out of phase with that in South Africa. In the seasonal A(H1N1) analysis, Antananarivo clustered with Uganda, while South Africa represented its own group.

A(H3N2) circulation in Antananarivo seemed to be entirely decoupled from that in Northern Hemisphere countries, as it led (2005, 2006, and 2008), coincided (2004 and 2007), or lagged (2002, 2003, 2009, 2011 to 2012) these countries depending on the year (Figure4). Further, the irregular pattern of A(H3N2) circulation in Antananarivo did not align with the marked winter-seasonal cycles of this virus in South Africa. In fact, there was no obvious temporal overlap of A(H3N2) in Antananarivo with any of the other studied countries, with Antananarivo representing its own singleton in hierarchical tree (Figure4).

Pandemic A(H1N1)pdm09 virus activity peaked in October 2009 in Antananarivo, a few weeks later than the second pandemic wave in the USA and the UK. The timing of pandemic activity in Antananarivo broadly coincided with the main 2009 pandemic wave in Italy, Switzerland, Germany, and France, and these five locales formed a cluster representing late pandemic activity (Figure4). Most of the African countries studied experienced earlier pandemic activity than Antananarivo, and the delay was especially pronounced with South Africa and La Réunion. In the period after 2009, circulation of A(H1N1)pdm09 was rare in Antananarivo and did not particularly coincide with any other country.

Finally, influenza B activity did not show a consistent seasonal pattern in Antananarivo, with two peaks in 2002, and large epidemics in 2007, 2009, 2012 (Figure4). Influenza B activity in Antananarivo was asynchronous with that of other locations so that Madagascar clustered as a singleton, separated from the Europe–US group and another group representing all other African countries.

### Climatology

**Figure 5:**
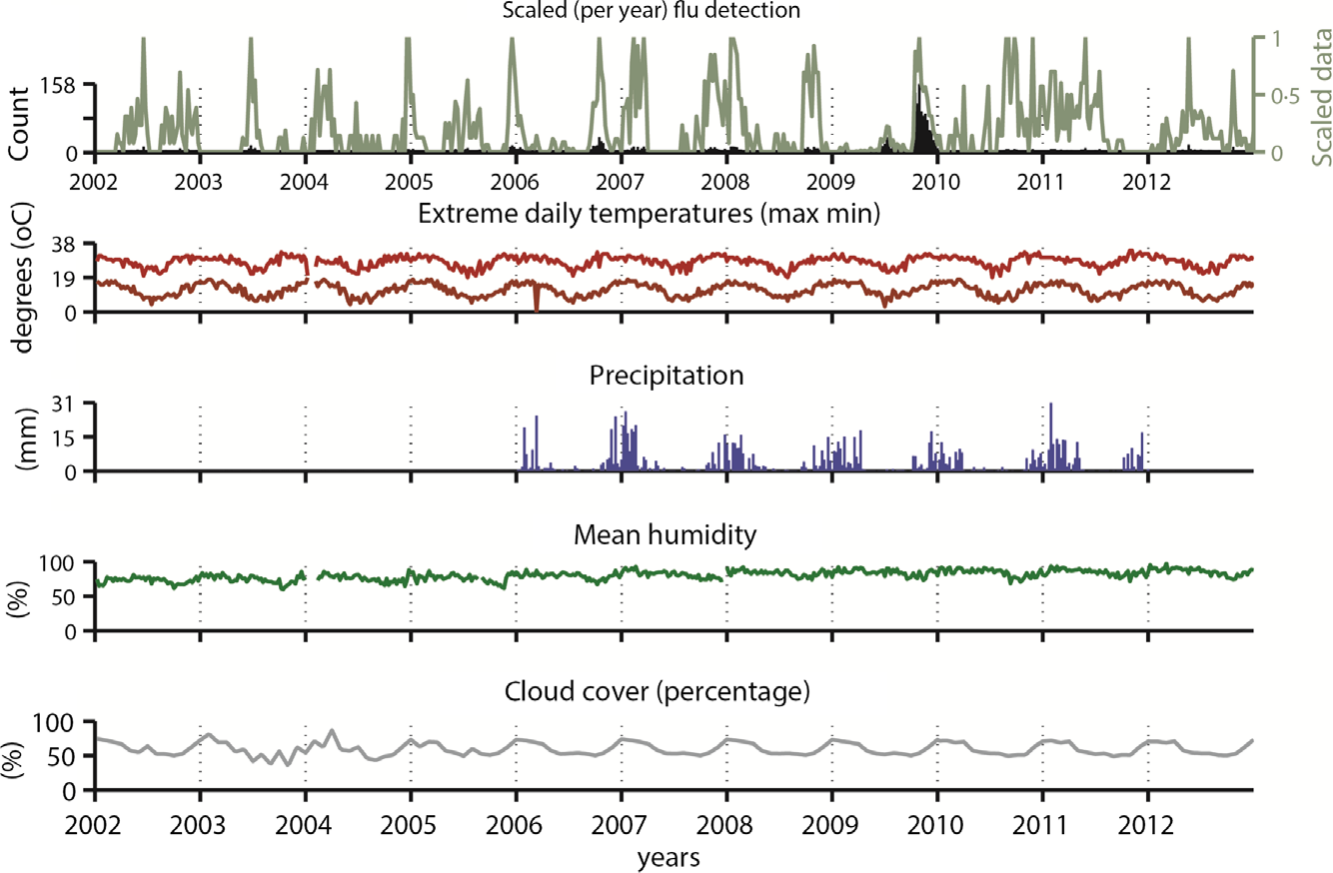
Weekly total influenza activity and climatic time series in Antananarivo, Madagascar, 2002–2012.

Influenza activity in Antananarivo was positively correlated with all climatic variables studied, but only weakly (Spearman correlation coefficient ≤ 0·22) (Figure5). Precipitation was the variable which presented the highest correlation linear correlation (0·22), found at lag zero. However, in the 5 years included in the analyses for this variable, viral circulation precedes the onset of rain in at least 2 years (e.g., years 2006 and 2010 in Figure S2). It is clear that influenza does not circulate preferentially in the colder months in Antananarivo (in fact, it would be the opposite given the sign of the correlation).

## Discussion

Our analyses indicate that the timing of influenza activity in Antananarivo is greatly irregular between years and is not driven by influenza circulation in countries that contribute most visitors to Madagascar. In fact, influenza circulation in Antananarivo precedes that in France in several studied years, although the greatest influx of visitors is from France, and by a large margin. Further, influenza activity in Antananarivo does not coincide with travel season, which peaks in July, and does not correspond to a preferred period of influenza activity. Our comparison of influenza activity in Antananarivo with that of other relevant locales reveals different epidemiologically relevant groupings by virus subtype, with Antananarivo clustering either on its own (for influenza B and A(H3N2)), with Uganda (for seasonal A(H1N1)), with DRC, Mauritius and Uganda (for all influenza viruses), or with European countries that experienced late 2009 pandemic activity (for A(H1N1)pdm09).

Interestingly, influenza circulation in Antananarivo is not synchronous with that in South Africa, a geographically close country with robust influenza surveillance and marked wintertime influenza seasonality.^30^ In fact, although Antananarivo usually experiences two irregular influenza peaks in the year (roughly one in the middle of each semester), these typically straddle the Southern and Northern Hemisphere winter seasons. We did not detect statistically significant influenza associations between Antananarivo and the sub-Saharan African countries studied, although Kenya (and possibly Mauritius) had a similar pattern of year-round circulation with rather ill-defined epidemic cycles. Rwanda and DRC have somewhat clearer seasonal cycles peaking in March–May, although low sampling size and a short surveillance period hamper robust comparisons. The most intriguing perhaps was that La Réunion had rather well-defined epidemic periods peaking in October–November, in-between the Southern and Northern Hemisphere seasons, which contrasts with the lack of influenza seasonality identified in Antananarivo.

We did not find any strong association between climatic variables and influenza activity in Antananarivo. The highest correlation was with precipitation, in line with expected patterns in the Tropics, although the association remained weak (ρ = 0·22), and influenza activity preceded the onset of the rainy season in some years in Antananarivo. Unfortunately, precipitation was the climatic variable with shortest records (5 years). We repeated the analysis with a longer precipitation dataset available for the entire study period on a monthly basis and still found weak correlation with influenza (ρ < 0·17). Due to its high altitude, Antananarivo experiences a mild subtropical climate and it is possible that limited climate fluctuations throughout the year do not lead to a preferred period of influenza activity. Specific humidity has been proposed as an important driver of influenza activity in temperate regions,^15^ and a recent global climate model indicates that regions where specific humidity falls below a threshold of 12 g/kg are predicted to have influenza peaks in cool and dry months.^7^ Humidity falls slightly below this threshold for most of Madagascar, except for the wet western coastal areas ((Figure S3), and hence, influenza activity is predicted to peak in cool and dry months of the year in Antananarivo,^7^ which runs contrary to our findings. These conflicting results echo those of a recent Chinese study, which suggests that influenza seasonality may not obey environmental forcing in mid-latitude regions, and could be driven by population mobility or local factors that have yet to be identified.^28^ Overall, the timing of influenza activity in Antananarivo appears to obey unidentified and irregular factors and does not follow patterns in locales in close contact and/or geographic proximity.

There is great interest in gaining a better understanding of the global circulation of influenza viruses, especially in interpandemic periods. Early research has put forward the concept of a sink–source model for A(H3N2) that favors the emergence of new virus variants in tropical South-East Asia.^31^,^32^ This model is still debated, in part due to the paucity of influenza epidemiological and virological information from Africa and South America.^33^ A further issue is the seemingly lack of persistence and low diversity of influenza viruses in tropical locations such as Hong Kong, which is inconsistent with the role of “source population”.^34^ A recent phylogenetic analysis has evidenced unusual persistence of A(H1N1)pdm09 virus in West Africa,^35^ which, combined with the particularly late arrival of the pandemic virus in this region,^36^ suggests that parts of Africa may be lagging in the global circulation of viruses. Our analysis indicates that Madagascar may be moderately well connected to global influenza circulation, as the island experienced a wave of pandemic activity synchronous with that of continental Europe, but seasonal influenza activity is not typically linked with that in any other country. Phylogenetic analyses similar to ones performed by Bahl *et al*. and Nelson *et al*. 34,35 would help quantify the extent of viral migrations between Madagascar and other regions and refine our understanding of global influenza virus circulation.

Our study has several limitations. First, we did not have precise information on influenza antigenic or genetic composition because unfortunately WHO FluNet does not provide publically available information beyond the subtype (seasonal A(H1N1), A(H1N1)pdm09, A(H3N2), or influenza B). More resolved data could help provide guidance on which of the Northern or Southern Hemisphere vaccine recommendations would be most appropriate for Madagascar. This is not an obvious choice for tropical countries, as illustrated by important variation in influenza seasonality throughout large countries such as Brazil 9 and China.^28^ Moreover, we did not attempt to apply phylogenetic methods to infer local persistence and precise viral migrations patterns between different locales, which would be an interesting area for future studies if sampling is strengthened.^31^,^32^,^35^,^37^ Finally, we did not have information from coastal areas of Madagascar which experience different climate from the capital city. A head-to-head comparison of influenza seasonality throughout the island would be worth pursuing as influenza surveillance strengthens nationwide.

We also had to face the limitations of limited sampling not only from Madagascar but also from the region. Indeed, neighboring countries such as Comoros, Mauritius, and La Réunion have intense connections to Madagascar but no or little available data. For example, La Réunion Island has an existing influenza surveillance system, but data were aggregated with those of mainland France before 2009 and publicly unavailable. The situation improved after the 2009 pandemic, and the ensuing boost in virological surveillance has already being extremely useful in describing and understanding the pandemic in this country.^25^,^38^–^40^ Nevertheless, lack of data availability prior to 2009 certainly diminishes the reliability of any time series analysis, including exploring associations with putative climatic drivers. Interestingly, years following the pandemic were of very low (and sparsely distributed through the year) influenza activity in Madagascar and elsewhere, perhaps due to an important depletion of susceptible in the post-pandemic period. Finally, to assess the link between climatic factors and influenza circulation, our analysis relies solely on Spearman correlation coefficient. We did not test other alternative methods including multivariate analyses (stepwise selection, dynamic regression models, etc.) because of potential multicollinearity among variables (a critical issue among climatic variables). Indeed, we considered that transparency and simplicity were valuable features of our exploratory study. The choice of Spearman correlation (as opposed to Pearson) was pertinent because it is a nonparametric measure based on less stringent assumptions about the data.

In conclusion, influenza activity in Madagascar lacks synchrony with that of geographically close or well-connected locales and is not driven by climate, indicating that we still lack reliable biological hypotheses to explain the seasonality of influenza in mid-latitude regions. Future studies in this country and other regions where influenza seasonality is ill-defined are needed to fully resolve the global drivers of influenza circulation, as well as that of other respiratory viruses. Currently, Madagascar lacks vaccine recommendations specific to the local epidemiological situation, so that the country is using the Northern Hemisphere vaccine formulation available from November to February. Moreover, vaccine coverage remains very low to inexistent even among at-risk group. Therefore, it is difficult to address question related to vaccine efficacy or relevance in Madagascar. Thus, deciphering the local drivers of influenza activity may help health authorities identify the optimal timing(s) and formulation of vaccines for routine immunization.

## Supporting Information

**Figure S1.** Monthly number of non-resident visitors to Madagascar, 2002–2012.

**Figure S2.** Scaled (per year) influenza detection (in red) and precipitation (in blue). Precipitation is the variable which presented a higher correlation (ρ = 0·22) with influenza detection. Here they are plotted in semi-overlapped axis to facilitate visual inspection. We did not enter in this analysis data from year 2009 (the pandemic year) and years for which precipitation data were not available (from 2002 to 2005), but these data are showed in pale colors.

**Figure S3.** Map of minimum specific humidity (g/kg) in Madagascar and the broader region. Past work has predicted that influenza activity may coincide with the cool and dry season in locations where specific humidity goes below a threshold of 12 g/kg (blue shaded areas) and with the rainy season in locations that remain above this threshold year-round. The 12 g/kg humidity threshold is barely crossed in Antananarivo.
