# Supplementary figures and images for: Influenza seasonality in Madagascar: the mysterious African free-runner
Source: Influenza Other Respir Viruses. 2015 Apr 23;9(3):101–9. doi: 10.1111/irv.12308 (PMC4415694; doi:10.1111/irv.12308)

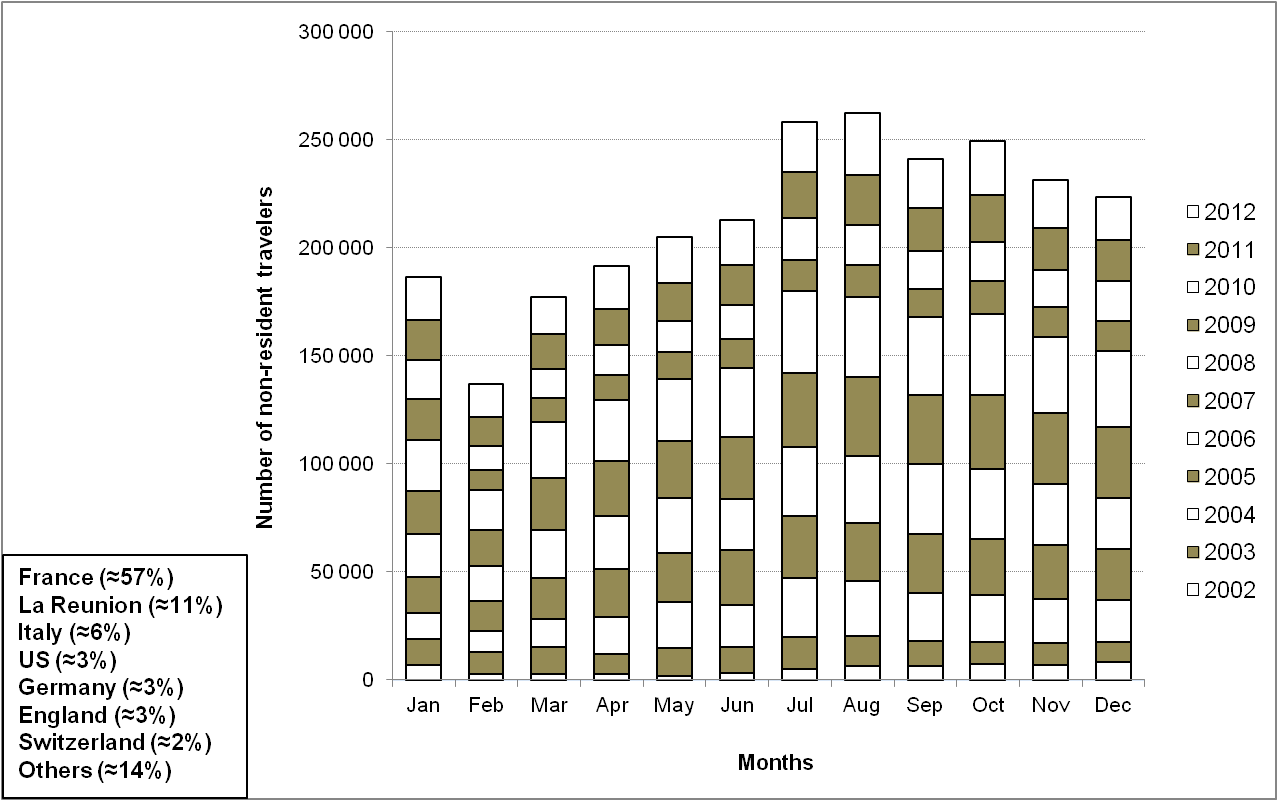

Supplement: Supplementary file 1 [file irv0009-0101-sd1.tif]

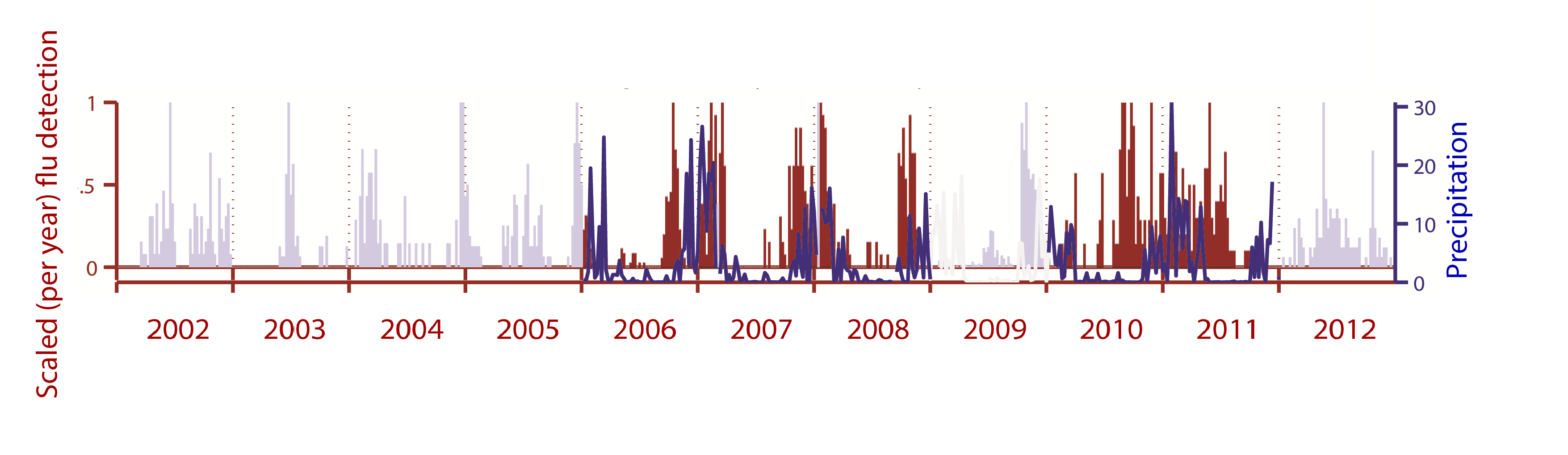

Supplement: Supplementary file 2 [file irv0009-0101-sd2.tif]

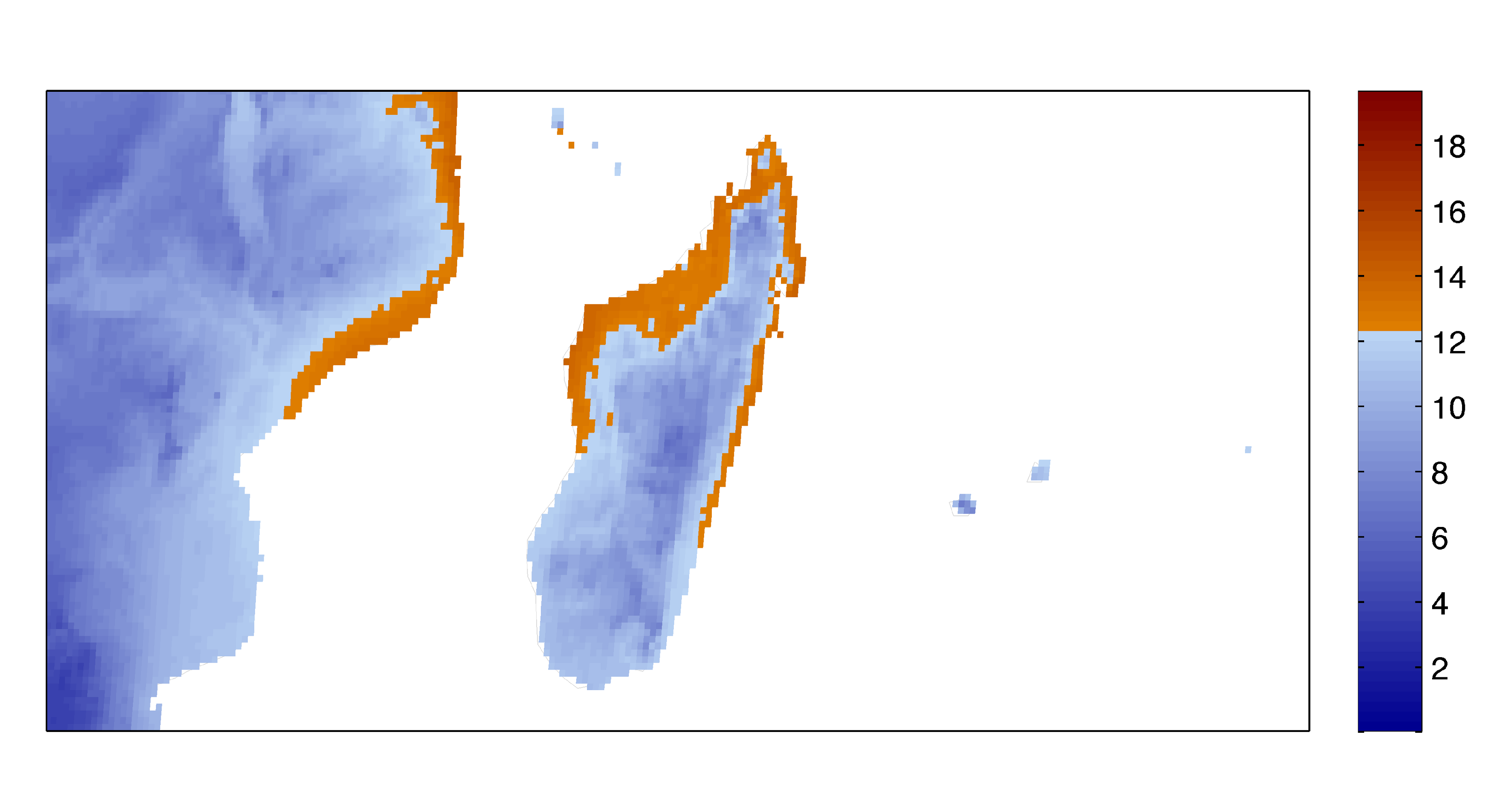

Supplement: Supplementary file 3 [file irv0009-0101-sd3.tif]
